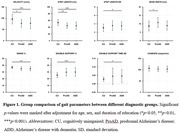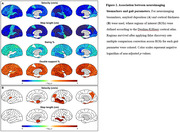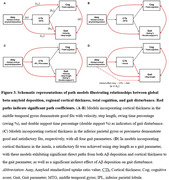# Mechanistic links of amyloid, atrophy, and cognition profiles for gait disturbance in Alzheimer's disease

**DOI:** 10.1002/alz.090225

**Published:** 2025-01-09

**Authors:** Min Seok Baek, Joon‐Kyung Seong, Sung‐Woo Kim, Dong Ho Kim, Jin Yong Hong

**Affiliations:** ^1^ Wonju Severance Christian Hospital, Yonsei University Wonju College of Medicine, Wonju Korea, Republic of (South); ^2^ Korea University, Seoul Korea, Republic of (South)

## Abstract

**Background:**

This study delves into the relationship between gait disturbances and the progression of Alzheimer's disease (AD), focusing on how these changes correlate with cognitive impairments and key neuropathological indicators.

**Method:**

We prospectively enrolled 48 patients with AD dementia (ADD), 27 patients with prodromal AD (proAD), and 41 cognitively unimpaired (CU) individuals between January 2022 and May 2023. Participants underwent brain T1‐weighted MRI, ^18^F‐florbetaben PET, neuropsychiatric tests, and APOE genotyping, and quantified gait analysis was assessed using a 5.79‐m long pressure sensor embed walkway. To evaluate the relationships between gait parameters, cognitive scores, regional amyloid deposition, and regional cortical thickness, Pearson partial correlation coefficients were computed, adjusting for age, sex, education duration, and leg length. Path analysis was employed to test hypothetical models comprising cortical amyloid deposition, cortical thickness, cognition, and gait parameters. Our models hypothesized five main pathways: from amyloid deposition to cortical thickness, cognition, and gait parameters; and from cortical thickness to cognition and gait parameters.

**Result:**

As AD progresses, a significant decline in velocity (p=0.0144 for comparing CU and proAD; p=0.0010 for comparing CU and ADD) and step length ( p=0.0033 for comparing CU and proAD; p= 0.0002 for comparing CU and ADD) was observed, indicative of a slower gait pace. The swing time percentage was reduced, and the double support time percentage was increased, showing a significant difference only in ADD patients ( p=0.0015 and p=0.0007 respectively). Gait variability, reflected in the standard deviations of step length and double support time, escalated with AD progression ( p=0.0173 and p=0.0143 for comparing CU and ADD, respectively) (Figure 1). Changes in gait pace, variability, and rhythm were found to be linked to neuroimaging markers of AD, including Aβ deposition in widespread areas and cortical atrophy in the inferior parietal and middle temporal gyri, precuneus, and insula (Figure 2). Path analysis revealed directed relationships between Aβ deposition and cognitive impairment, as well as cortical atrophy, collectively impacting gait disturbance in AD (Figure 3).

**Conclusion:**

Our analysis indicates directed relationships between amyloid deposition and cognitive impairment, as well as cortical atrophy in these regions, collectively impacting gait disturbance in AD